# Suicidal ideation in medical students of Hebei province: prevalence and associated factors

**DOI:** 10.3389/fpsyt.2024.1398668

**Published:** 2024-07-30

**Authors:** Fanfan Huang, Wenting Lu, Xiaochuan Zhao, Na Li, Tianyu Zhao, Shijie Guo, Bufan Liu, Ruojia Ren, Li Yang, Lan Wang, Yuanyuan Gao, Ran Wang, Mei Song, Quan Lin, Yuehang Xu, Na Jin, Kuaikuai Liu, Meina Bai, Xueyi Wang

**Affiliations:** Mental Health Center, The First Hospital of Hebei Medical University, Hebei Clinical Medical Research Center for Mental Disorders, Shijiazhuang, China

**Keywords:** suicidal ideation, medical students, prevalence, risk factors, childhood maltreatment, stressful life events

## Abstract

**Objectives:**

This study investigated the prevalence of suicidal ideation (SI) among Chinese medical students and its associated risk factors.

**Methods:**

A total of 6643 medical students (2383 males/4260 females) were recruited from a medical college in Hebei Province, China. Demographic data were collected via a self-administered questionnaire. The Childhood Trauma Questionnaire Short Form (CTQ-SF) was used to evaluate childhood maltreatment (CM), and the Adolescent Self-Rating Life Events Checklist (ASLEC) was used to evaluate the stressful life events. Suicidal ideation was assessed using the Beck Scale for Suicide Ideation (BSSI). Univariate and multivariate logistic regression models were used to analyze the factors affecting SI.

**Results:**

The prevalence of SI in medical students was 11.5% (763/6643). Multivariate logistic regression analysis revealed that SI was significantly associated with younger age, a female sex, being lovelorn, being introverted, experiencing CM during childhood, and experiencing stressful life events within the past 12 months. Of the five subtypes of CM, emotional abuse may have the strongest effect on SI (OR=2.76, 95% CI: 1.72–4.42). The joint effects of CM and stressful life events were significantly associated with an increased risk of SI (OR=5.39, 95% CI: 4.15–6.98).

**Conclusion:**

The prevalence of SI among medical students is high, and medical students who have experienced CM and stressful life events have a higher tendency towards SI. Screening for both CM and stressful life events may be an effective way of identifying individuals at high risk of SI.

## Introduction

Suicide is a critical public health concern and the foremost cause of death among people 15–24 years of age (1). The suicide model emphasizes the successive developmental stages of suicidal behavior: suicidal ideation (SI), suicidal plan, suicidal attempt and finally the completion of suicide (2, 3). Based on this theory, SI can be viewed as a sign of subsequent suicidal behavior; the risk of suicide gradually develops from being mild to being high. Suicidal ideation refers to an individual’s willingness to end his or her life by direct or indirect means (4). Although SI does not always lead to death, SI is a major link in the development of fatal suicidal behavior (5). It has been shown that roughly 10% of Chinese adolescents have SI (6); adolescents with SI exhibit a significantly higher risk of suicide (7). Therefore, it is critically important to explore the risk factors of SI with the goal of developing targeted suicide-prevention programs.

Suicidal ideation in adolescents is caused by a complex interaction of physiological and psychosocial factors. Previous studies have demonstrated a significant association between childhood maltreatment (CM) and suicide (8, 9). Childhood maltreatment is typically defined as a significant negative experience in early life. It generally encompasses five subtypes: emotional abuse (EA), emotional neglect (EN), physical abuse (PA), physical neglect (PN), and sexual abuse (SA) (10). The risk of suicide increases by anywhere from 2.7- to 6.1-fold with any type of CM (11). A study of a representative sample of adults (12) similarly found that CM was associated with a significant increase suicide risk in each developmental stage of a suicidal model. Furthermore, previous studies have focused more on the impact of CM on SI and ignored the impact of stressful life events on SI, which are more common and widespread than CM. Stressful life events—such as pressure to perform academically and stressors associated with interpersonal relationships—are associated with SI in college students (13, 14). Stressful life events are a major source of stress for SI, which can strongly predict SI (15) and play a triggering role in suicidal behavior (16). Both CM and stressful life events have a clear impact on SI, but the joint effects of CM and stressful life events on SI have not been explored.

The transition from adolescence to adulthood is an important period of psychological change. During this time, individuals explore their self-identity and values and increase their social responsibilities and expectations about their goals. The uncertainty that marks this period of time may cause adolescents to experience an uptick in mental health problems (17). Compared with non-medical students, medical students typically have a relatively heavy academic schedule, strict clinical work requirements, and they are furthermore exposed to stressors such as patient deaths. Medical students may accordingly have a higher risk of SI. According to previous research, the global prevalence of SI among medical students was 11.1% (18); the prevalence of SI among Chinese medical students was 17.9% (19). This can adversely affect the long-term health of medical students.

It is critical to explore the risk factors of SI in medical students. Although CM is widely recognized to be a strong predictor of SI, the impact of different subtypes of CM on SI remains controversial. In addition, there are no studies have investigated the impact of the joint effects of CM and stressful life events on SI in medical students. This study accordingly investigates the association between SI and different subtypes of CM in a large sample of Chinese medical students and explores the joint effects of CM and stressful life events on SI.

## Methods

### Study design and participants

The study was a cross-sectional design based on the Internet self-assessment questionnaire, and the research team members of this study served as investigators. The screening tool was an information network evaluation system developed by Saitron Information Co., Ltd., and the whole evaluation process took about 30 minutes. Participants had to be met the following inclusion criteria:(1)age ≥18 years;(2)willingness to sign the consent to participate form. All of the participants were in their sophomore, junior year or senior years of medical undergraduate study at a university in Hebei Province, China. Considering that the final exam may affect the mood of the participants, we recruited the subjects between June 15 and June 27, 2021. Prior to this time period, all participants had completed the final exam. At the same time, the damage caused to human beings by the COVID-19 is gradually decreasing under the natural law, and the reasonable epidemic prevention measures taken by the government have also put students’college life on the right track. Subsequently, the research team conducted a structured interview with all subjects using the Mini-International Neuropsychiatric Interview (MINI 5.0) between October 11 and November 7, 2021, to assess the prevalence of any mental disorders within 12 months.

We have harmonized survey methods and guidelines. First and foremost, all of the medical students who agreed to participate in the study were explained the purpose of the study and provided an official consent form. Second, all of the participants scanned the QR code and logged into the system using a virtual student number and their initials, in this way, the privacy of the participants is protected to the greatest extent. The participants filled in all the information in a quiet classroom, and the research team members of this study answered questions on site. Finally, all the data is sorted out by professional. Of the 7578 participants, 105 were excluded because they refused to participate or asked for leave; we additionally eliminated 297 questionnaires with incomplete data or logical contradictions; 533 were excluded because they had a history of mental disorders. The final sample size was 6643.

The study protocol was approved by the First Hospital of Hebei Medical University Ethics Committee (Code: 20210354).

### Assessment of CM

The Childhood Trauma Questionnaire Short Form (CTQ-SF) (20) consists of 28 items and measures childhood abuse and neglect experiences that can be categorized as falling into five subtypes: EA, PA, SA, EN, and PN. The participants were asked about the CM they experienced before turning 16. Subjects with CM were defined as having at least one of following: EA ≥13 points, PA ≥10 points, SA ≥8 points, EN ≥15 points, or PN ≥10 points (21). The Chinese version of CTQ-SF has good validity and reliability (22).

### Assessment of stressful life events

The Adolescent Self-Rating Life Events Checklist (ASLEC) was used to assess the negative life events within the past 12 months. The 27-item, Chinese version of the ASLEC developed by Liu (23) is a self-evaluation scale reflecting the physiological and psychological characteristics and social roles of adolescents. There are six dimensions: interpersonal relationships, learning stress, punishment, loss, health & adaptation, and others. Participants were asked if they had experienced any of these life events within the past 12 months. Responses to each item were coded as “yes” or “no” to indicate the presence or absence of the negative life events (no=0, yes=1). The validity and reliability of the ASLEC were proved to be good (24).

### Assessment of SI

We used the 19-item Beck Scale for Suicide Ideation (BSSI) (25) to measure the SI of the participants. The first five questions of this scale were used to assess the participants’ SI. If a participants answered “Weak”/“Moderate to strong” to the question “Desire to make active suicide attempt” or answered “Weak”/“Moderate to strong” to the question “Passive suicidal desire”, they were defined as a *suicidal ideator (SI)*. If not, they were defined as a non-suicidal ideator (NSI). For participants with SI, we took the necessary interventions. First of all, we told schools and guardians to strengthen care. Secondly, the research team provided a “green channel” for subjects with SI, that is, under the premise of the participants’ consent, they were provided with one-on-one psychological counseling services, provided psychological support. If necessary, participants are recommended for hospitalization, medication, physiotherapy, and psychotherapy. We also worked with the participants to develop a follow-up plan to prevent unexpected events.

### Demographic characteristics

General demographic data—age (years), gender (male/female), only-child status (yes/no), grade (sophomore/junior/senior), parental marital status (both parents/single parent), relationship status (in love/single/lovelorn) and personality characteristics (between introversion and extroversion/introversion/extroversion) were collected from all of the participants.

### Statistical analysis

Means (M) and standard deviations (SD) were used for continuous variables, and counts (n) and proportions (%) were used for the categorical variables. T-tests were used to compare the differences in the continuous variables across groups. Chi-square tests were used to analyze the differences in categorical variables across groups. Univariate and multivariate logistic regression analyses were used sequentially to explore associated factors of SI among all of the surveyed medical students. We also performed collinearity diagnostics between variables. Two-sided *p*-values <0.05 are statistically significant. Crude and adjusted odds ratios (ORs) and their 95% confidence intervals (CIs) were computed. All of the analyses were performed using SPSS (version 26.0).

## Results

### General characteristics of the study participants

The demographic characteristics of the participants are presented in **Table 1**. The average age of the participants was 21.0 (SD=1.0). Roughly two-thirds (64.1%) of the participants were female. The prevalence of SI was 11.5% among all of the participants. 22.0% of the participants reported at least one adverse childhood experience. Among the various subtypes of CM, 16.8% of the participants reported experiencing PN, 6.7% reported experiencing EN, 2.8% reported experiencing SA, 1.4% reported experiencing EA, and 1.3% reported experiencing PA. Participants with stressful life events accounted for 66.3% of the overall sample.

**Table 1:** Demographic and clinical variables of medical students (N=6,643).

Variables	Total (N=6643)	No suicidal ideation (N=5880)	Suicidal ideation (N=763)	*t*/*χ2*	*P* value
Age:year, mean (SD)	21.0 (1.0)	21.0 (1.0)	20.9 (1.0)	1.27	0.204
Gender: N (%)					
Male	2383 (35.9)	2140 (89.8)	243 (10.2)	6.07	0.014
Female	4260 (64.1)	3740 (87.8)	520 (12.2)		
Only-child: N (%)					
No	4614 (69.5)	4098 (88.8)	516 (11.2)	1.36	0.244
Yes	2029 (30.5)	1782 (87.8)	247 (12.2)		
Grade: N (%)					
Sophomore	3110 (46.8)	2756 (88.6)	354 (11.4)	0.06	0.969
Junior	2776 (41.8)	2455 (88.4)	321 (11.6)		
Senior	757 (11.4)	669 (88.4)	88 (11.6)		
Parental marital status: N (%)					
Both parents	6194 (93.2)	5505 (88.9)	689 (11.1)	11.82	0.001
Single parent	449 (6.8)	375 (83.5)	74 (16.5)		
Relationship status: N (%)					
In love	1759 (26.5)	1562 (88.8)	197 (11.2)	13.27	0.001
Single	4784 (72.0)	4241 (88.6)	543 (11.4)		
Lovelorn	100 (1.5)	77 (77.0)	23 (23.0)		
Personality characteristics: N (%)					
Between introversion and extroversion	4137 (62.3)	3691 (89.2)	446 (10.8)	28.73	<0.001
Introversion	1308 (19.7)	1104 (84.4)	204 (15.6)		
Extroversion	1198 (18.0)	1085 (90.6)	113 (9.4)		
Childhood maltreatment:					
Emotional abuse: N (%)					
No	6548 (98.6)	5826 (89.0)	722 (11.0)	95.10	<0.001
Yes	95 (1.4)	54 (56.8)	41 (43.2)		
Emotional neglect: N (%)					
No	6198 (93.3)	5547 (89.5)	651 (10.5)	87.83	<0.001
Yes	445 (6.7)	333 (74.8)	112 (25.2)		
Sexual abuse: N (%)					
No	6456 (97.2)	5731 (88.8)	725 (11.2)	14.77	<0.001
Yes	187 (2.8)	149 (79.7)	38 (20.3)		
Physical abuse: N (%)					
No	6554 (98.7)	5818 (88.8)	736 (11.2)	31.53	<0.001
Yes	89 (1.3)	62 (69.7)	27 (30.3)		
Physical neglect: N (%)					
No	5527 (83.2)	4956 (89.7)	571 (10.3)	43.15	<0.001
Yes	1116 (16.8)	924 (82.8)	192 (17.2)		
Stressful life events: N (%)					
No	2242 (33.7)	2122 (94.6)	120 (5.4)	125.22	<0.001
Yes	4401 (66.3)	3758 (85.4)	643 (14.6)		

We found statistically significant differences between the SI group and the NSI group in terms of gender (χ^2^ = 6.07, *p*=0.014), parental marital status (χ^2^ = 11.82, *p*=0.001), relationship status (χ^2 ^= 13.27, *p*=0.001), personality characteristics (χ^2^ = 28.73, *p*<0.001), EA (χ^2^ = 95.10, *p*<0.001), EN (χ^2^ = 87.83, *p*<0.001),SA (χ^2^ = 14.77, *p*<0.001), PA (χ^2^ = 31.53, *p*<0.001), PN (χ^2^ = 43.15, *p*<0.001) and stressful life events (χ^2^ = 125.22, *p*<0.001). There was no significant difference of age, only-child status and grade between the SI group and the NSI group (*p* >0.05).

### Associated factors of SI in medical students

Data from the logistic regression analyses are listed in **Table 2**. The Hosmer-Lemeshow test revealed that the model fit the data well (χ^2^ = 15.25, *p*=0.055). A female sex (OR=1.28, 95% CI: 1.08–1.52), a lovelorn status (OR=2.17, 95% CI: 1.31–3.61), being introversion (OR=1.48, 95% CI: 1.23–1.78), EA (OR=2.76, 95% CI: 1.72–4.42), EN (OR=1.81, 95% CI: 1.39–2.37), PN (OR=1.37, 95% CI: 1.12–1.66) and the occurrence of stressful life events (OR=2.76, 95% CI: 2.25–3.39) were all significantly associated with an increased risk of SI. However, among all five subtypes of CM, we did not find a correlation between PA (*p*=0.400), SA (*p*=0.161) and SI. In addition, the age (OR=0.89, 95% CI: 0.80–0.98) was inversely correlated with the risk of SI.

**Table 2:** Logistic regression analysis of suicidal ideation (N=6,643).

	Unadjusted OR (95% CI)	*P* value	Adjusted OR (95% CI)	*P* value
Age (+1yrs)	0.95 (0.88, 1.03)	0.204	0.89 (0.80, 0.98)	0.022
Gender : Female (Ref: Male)	1.22 (1.04, 1.44)	0.014	1.28 (1.08, 1.52)	0.005
Only-child:yes (Ref: No)	1.10 (0.94, 1.29)	0.244	1.17 (1.00, 1.39)	0.065
Grade (Ref: Sophomore)				
Junior	1.02 (0.87, 1.19)	0.828	1.19 (0.98, 1.44)	0.082
Senior	1.02 (0.80, 1.31)	0.851	1.20 (0.87, 1.66)	0.267
Parental marital status (Ref: Both parents)				
Single parent	1.58 (1.21, 2.05)	0.001	1.31 (0.99, 1.71)	0.056
Relationship status (Ref: In love)				
Single	1.02 (0.85, 1.21)	0.864	0.97 (0.81, 1.16)	0.764
Lovelorn	2.37 (1.45, 3.86)	0.001	2.17 (1.31, 3.61)	0.003
Personality characteristics (Ref: Between introversion and extroversion)				
Introversion	1.53 (1.28, 1.83)	<0.001	1.48 (1.23, 1.78)	<0.001
Extroversion	0.86 (0.69, 1.07)	0.180	0.84 (0.67, 1.05)	0.132
Emotional abuse:yes (Ref: No)	6.13 (4.05, 9.26)	<0.001	2.76 (1.72, 4.42)	<0.001
Emotional neglect:yes (Ref: No)	2.87 (2.28, 3.60)	<0.001	1.81 (1.39, 2.37)	<0.001
Sexual abuse:yes (Ref: No)	2.02 (1.40, 2.90)	<0.001	1.32 (0.89, 1.96)	0.161
Physical abuse:yes (Ref: No)	3.44 (2.18, 5.44)	<0.001	1.25 (0.74, 2.12)	0.400
Physical neglect:yes (Ref: No)	1.80 (1.51, 2.16)	<0.001	1.37 (1.12, 1.66)	0.002
Stressful life events:yes (Ref: No)	3.03 (2.47, 3.70)	<0.001	2.76 (2.25, 3.39)	<0.001

OR, odds ratio.

### CM, stressful life events, and SI

**Table 3** presents data about the impact of the joint effects of CM and stressful life events on SI in our medical-student cohort. The impact of CM and stressful life events on SI were divided into the following four types: (1) Without CM, without stressful life events; (2) Without CM, with stressful life events; (3) With CM, without stressful life events; (4) With CM, with stressful life events. Compared with medical students who experienced neither CM nor stressful life events, students with stressful life events and without CM had a 2.81-fold higher risk of SI (OR=2.81, 95% CI: 2.22–3.57); medical students with CM and without stressful life events had a 2.04-fold higher risk of SI (OR=2.04, 95% CI: 1.33–3.14). We also found that the risk of SI was 5.39-fold higher among medical students with CM and stressful life events (OR=5.39, 95% CI: 4.15–6.98).

**Table 3:** Associations between childhood maltreatment, stressful life events, and suicidal ideation (N=6,643).

	No SI		SI			
	n	%	n	%	Unadjusted OR	Adjusted OR^a^
Without CM, without stressful life events	1813	95.3	89	4.7		
Without CM, with stressful life events	2875	87.7	405	12.3	2.87 (2.26, 3.64)	2.81 (2.22, 3.57)
With CM, without stressful life events	309	90.9	31	9.1	2.04 (1.34, 3.13)	2.04 (1.33, 3.14)
With CM, with stressful life events	883	78.8	238	21.2	5.49 (4.25, 7.10)	5.39 (4.15, 6.98)

a Adjusted for age, gender, grade, only-child, parental marital status, relationship status, personality characteristics. OR, odds ratio; CM, childhood maltreatment; SI, suicidal ideation.

## Discussion

This study investigated the prevalence of SI and its associated risk factors at a medical college in Hebei Province, China. There are several major findings. First, the prevalence of SI among medical students is high. Second, both CM and stressful life events are independently associated with the risk of SI. Third, the joint effects of CM and stressful life events are significantly associated with an increased risk of SI. Our findings highlight that screening for both CM and stressful life events may be an effective way of identifying individuals at high risk of SI.

### High prevalence of SI in medical students

We found that the prevalence of SI in medical students was 11.5%, which was higher than the rate reported among other college students in China (7.5%) (26). As mentioned above, the unique nature of medical majors may cause medical students to have more mental stress than non-medical students. Other studies have reported that roughly 10% of medical students experience SI in their lifetime (18, 27), which is similar to our findings. However, some studies (19, 28) have found that the prevalence of SI in medical students is higher than the results that we found. Possible reasons for the difference include study sample size, measurement methods, and cultural differences. For example, some studies only relied on the question “Have you ever had thoughts of committing suicide?”to indicate SI. In our study we used the BSSI scale; this scale has high reliability and validity, and it can more accurately assess the SI of an individual (29).

### Associated factors of SI

The significant clinical correlates of SI in our study included younger age, a female sex, being lovelorn, and being introverted. Some studies have shown that females report significantly higher intensity of SI compared to males (30–32). Our study further proves that female students are more likely to have SI than the male. This finding may be related to the fact that the differences in Chinese parenting styles, Chinese families generally believe that more care should be given to the upbringing of girls, and there will be stricter discipline and requirements for boys, so girls’ psychological endurance and resilience will be lower than boys, and girls are more likely to have SI when they encounter adverse events in life (33). Furthermore, neuroendocrine changes that occur in different genders during adolescence may be at play (34). We also found that being lovelorn was at a greater risk of SI. One possible explanation for this finding is when they lack the experience to deal with emotional events, once they have a painful emotional experience, it often brings them psychological wounds that are difficult to heal and promotes SI. Our study found that introversion was a factor that increased the risk of SI, which also aligns with the previous finding that an American study demonstrated that individuals with lower levels of extroversion were more likely to experience SI (35). Introverted personality traits tend to manifest as poorer social interactions, higher levels of feeling loneliness, and lower social connections (36). This may be due to their more sensitive nature, which makes them more susceptible to interpersonal or other problems.

Our study found that all forms of CM, with the exception of PA and SA, had an effect on SI. EA was the strongest predictor of an increased risk of SI among five subtypes of CM. Negative values may be one of the cognitive outcomes of CM (37, 38), and negative values can motivate individuals to react negatively when they experience stress. Previous findings have supported links between CM and suicidal behavior (39, 40). Study has also shown that exposure to traumatic situations during childhood can affect an individual’s proper neurobiological and affective development. EA was the strongest predictor of emotion deregulation (41). CM can lead to negative coping styles and pessimistic worldviews, and indirectly increase the likelihood of suicidal behavior by increasing the risk of impulsivity and hopelessness (42, 43). However, we did not find an association between PA and SI. This result may be related to changes in traditional Chinese parenting styles, which today often replaces corporal punishment with vocal admonishment. No significant association between SA and SI were observed in this study. It was distinctive to the study by Bruffaerts et al., in which SA was the strongest risk factor for the onset of suicidal behaviour, especially during adolescence (44). Differences in study subjects, degree of exposure to the SA experience, or sample size may also contribute to the different results.

### The joint effects of CM and stressful life events

We also analyzed the joint effects of CM and stressful life events in relation to SI. Our study found that the joint effects of CM and stressful life events are significantly associated with an increased risk of SI; this finding still remained true after adjusting for confounders. Our finding supports the “cumulative stress hypothesis”, which suggests that neuropsychiatric disorders are largely triggered by the accumulation of multiple major adverse events (45). The continued accumulation of adverse experiences in childhood and adulthood is even more damaging (46). As our research found, the joint effects of CM and stressful life events significantly increase the risk of SI in individuals. The possible reason for this is that early exposure to CM increases an individual’s vulnerability to the effects of later stressful life events. Specifically, the experiences of CM in childhood can reduce an individual’s tolerance to relatively minor stressors, making the individual “sensitive”, which is called stress sensitization effects (47). Findings from our study highlight the need to continually evaluate the mental health status of medical students over time and provide them with appropriate mental health support and mental health services.

### Limitations

Several possible limitations to this study should be acknowledged. First, since CM was recalled, there is the potential for recall bias. Second, given that this investigation was a cross-sectional study we could not directly explain the causal relationship between SI and risk factors based on our results. Third, the study, which investigated SI among medical students in Hebei Province, China, has not yet been generalized nationwide, it should be prudent when interpretation and extrapolating our findings. Fourth, other potential contributing factors to SI were not taken into consideration, such as socioeconomic level and family history of mental disorders. Lastly, we did not know exactly when the CM occurred, the impact of CM on SI may vary according to the age at which the maltreatment occurred. Therefore, we need more research to validate our results.

## Conclusion

Suicide is the result of an accumulation of risk factors over a lifetime. Suicidal ideation can be considered to be an early warning sign of suicidal behavior, exploring the risk factors for SI is essential for early and effective interventions. This study found that the prevalence of SI was 11.5% among Chinese medical students. Higher rates of SI were associated with younger age, being female, being lovelorn, being introverted, experiencing maltreatment during childhood and stressful life events within the past 12 months. In addition, our findings highlight the potential joint effects of CM and stressful life events on an increased risk of SI. It is critical to screen individuals for CM and stressful life events to identify people at a high risk of SI. Suicide interventions could include reducing adverse childhood experiences and strengthening adaptive coping to stressful life events.

## Data availability statement

The original contributions presented in the study are included in the article/supplementary material. Further inquiries can be directed to the corresponding author.

## Ethics statement

The studies involving humans were approved by the First Hospital of Hebei Medical University Ethics Committee (Code: 20210354). The studies were conducted in accordance with the local legislation and institutional requirements. The participants provided their written informed consent to participate in this study.

## Author contributions

FH: Conceptualization, Data curation, Formal analysis, Investigation, Methodology, Project administration, Validation, Writing – original draft, Writing – review & editing. WL: Conceptualization, Data curation, Formal analysis, Investigation, Methodology, Project administration, Validation, Writing – original draft, Writing – review & editing. XZ: Data curation, Investigation, Validation, Writing – original draft. NL: Data curation, Investigation, Validation, Writing – original draft. TZ: Data curation, Investigation, Validation, Writing – original draft. SG: Data curation, Investigation, Validation, Writing – original draft. BL: Data curation, Investigation, Validation, Writing – original draft. RR: Data curation, Investigation, Validation, Writing – original draft. LY: Data curation, Investigation, Validation, Writing – original draft. LW: Data curation, Investigation, Methodology, Validation, Writing – original draft. YG: Data curation, Investigation, Methodology, Validation, Writing – original draft. RW: Data curation, Investigation, Methodology, Validation, Writing – original draft. MS: Data curation, Investigation, Methodology, Validation, Writing – original draft. QL: Data curation, Formal analysis, Investigation, Writing – original draft. YX: Data curation, Formal analysis, Investigation, Writing – original draft. NJ: Data curation, Formal analysis, Investigation, Writing – original draft. KL: Data curation, Formal analysis, Investigation, Writing – original draft. MB: Data curation, Formal analysis, Investigation, Writing – original draft. XW: Conceptualization, Data curation, Formal analysis, Funding acquisition, Methodology, Project administration, Resources, Software, Supervision, Visualization, Writing – review & editing.
